# Patterns of Cereal Yield Growth across China from 1980 to 2010 and Their Implications for Food Production and Food Security

**DOI:** 10.1371/journal.pone.0159061

**Published:** 2016-07-12

**Authors:** Xiaoyun Li, Nianjie Liu, Liangzhi You, Xinli Ke, Haijun Liu, Malan Huang, Stephen R. Waddington

**Affiliations:** 1 College of Economics and Management, Huazhong Agricultural University, Wuhan, Hubei, 430070, China; 2 International Food Policy Research Institute (IFPRI), 2033 K Street, NW, Washington DC, 20006, United States of America; 3 College of Land Management, Huazhong Agricultural University, Wuhan, Hubei, 430070, China; 4 College of Life Science and Technology, Huazhong Agricultural University, Wuhan, Hubei, 430070, China; 5 Apartado Postal 4–205, Cuernavaca, Morelos, CP62451, México; Tennessee State University, UNITED STATES

## Abstract

After a remarkable 86% increase in cereal production from 1980 to 2005, recent crop yield growth in China has been slow. County level crop production data between 1980 and 2010 from eastern and middle China were used to analyze spatial and temporal patterns of rice, wheat and maize yield in five major farming systems that include around 90% of China's cereal production. Site-specific yield trends were assessed in areas where those crops have experienced increasing yield or where yields have stagnated or declined. We find that rice yields have continued to increase on over 12.3 million hectares (m. ha) or 41.8% of the rice area in China between 1980 and 2010. However, yields stagnated on 50% of the rice area (around 14.7 m. ha) over this time period. Wheat yields increased on 13.8 m. ha (58.2% of the total harvest area), but stagnated on around 3.8 m. ha (15.8% of the harvest area). Yields increased on a smaller proportion of the maize area (17.7% of harvest area, 5.3 m. ha), while yields have stagnated on over 54% (16.3 m. ha). Many parts of *the lowland rice and upland intensive sub-tropical farming systems* were more prone to stagnation with rice, *the upland intensive sub-tropical system* with wheat, and maize in *the temperate mixed system*. Large areas where wheat yield continues to rise were found in *the lowland rice and temperate mixed systems*. Land and water constraints, climate variability, and other environmental limitations undermine increased crop yield and agricultural productivity in these systems and threaten future food security. Technology and policy innovations must be implemented to promote crop yields and the sustainable use of agricultural resources to maintain food security in China. In many production regions it is possible to better match the crop with input resources to raise crop yields and benefits. Investments may be especially useful to intensify production in areas where yields continue to improve. For example, increased support to maize production in southern China, where yields are still rising, seems justified.

## Introduction

Between 1980 and 2010, world cereal production increased by around 60% (land area decreased by 3.1% and yields per hectare rose by 65.3%) [1]. To meet projected global demand for food by 2050, agricultural crop production must increase by a further 50 to 60% from current levels [2,3]. Food production will also have to rise significantly in China (by an estimated 8.3% between 2010 and 2020) to address projected increases in human population and improvements in daily diet [4]. Despite China's strong economic performance and steady reduction in poverty, meeting that substantial increase in food demand without major damage to the environment will be a huge challenge. After a period of remarkable growth in China’s crop production, where cereal production rose by 86% between 1980 and 2005 [5], crop yield growth has slowed down recently. Current national cereal yields appear to have stagnated at around 6.2–6.7 t ha^-1^ for rice, 4.5–4.7 t ha^-1^ for wheat and 5.1–5.5 t ha^-1^ for maize [6]. Those three crops accounted for 80% of total crop area in China, and represent about 20% of global cereal production. However, there is substantial variation in yield performance across China, with some regions having reported yield declines and others continued improvement.

Some research is available that focuses on spatial changes to cereal crop area and regional patterns of crop production in China. The spatial changes in rice, wheat and maize production at a provincial scale in China during 1981–2008 and the factors that influenced the changes revealed some major spatial changes over this time period. Rice production increased rapidly in middle and northeast China while the proportion of rice area in North China and East China decreased. Chemical fertilizer inputs, agricultural machinery, effective irrigation, flood disaster and drought area were important factors that affected regional production [7]. A spatial production allocation model was used to develop a series of spatial distributions of rice area and production and found that the center of rice production has moved northeastward faster than rice area because of the significant increase in rice yields in Northeast China [8]. Overall, the center of cereal production has shown a tendency to move towards northern China [9,10]. A recent detailed study analyzed the trends of cereal crop yield and cultivated area at the county level throughout China from 1980 to 2008 [11]. Their findings show that yield stagnation is widespread (on almost half the area) for rice, wheat and maize in many major cereal production zones, and that closing yield gaps should be high priority. To help identify opportunities to close yield gaps and raise cereal production in China requires a study that focuses on the areas where these crops are mostly grown and where yields are increasing or have stagnated. This can then be used to explore the prospects for further yield improvements through intensification in those areas.

To assess the likelihood of meeting future cereal crop production needs in China, we undertook a study of yield trends over the period 1980 to 2010 for the three most important cereals (rice, wheat and maize) across the most important provinces and farming systems where those crops are grown. To do this we employed new long-term crop production data at county level and developed high resolution geospatial databases of Chinese cereal-based farming systems (identified from the FAO farming systems framework, see www.fao.org/farmingsystems), using thirty years of census bureau data for 2,463 counties. From these databases, we analyzed spatial and temporal patterns of yield change for rice, wheat and maize and determined locations where those crops have experienced increasing yields, and where crop yields have stagnated or declined. We discuss implications for cereal demand and examine ways of raising supply and yields through addressing constraints to production, including climate change, limited arable land and property rights, water shortages, fertilizer use and pollution. The findings from our analysis should improve the understanding of changes in cereal crop production and yield across the major food producing areas of China, and suggest areas for further investment in inputs and support to ensure future cereal production and food security.

## Methods

### Data development

**Fig 1:**
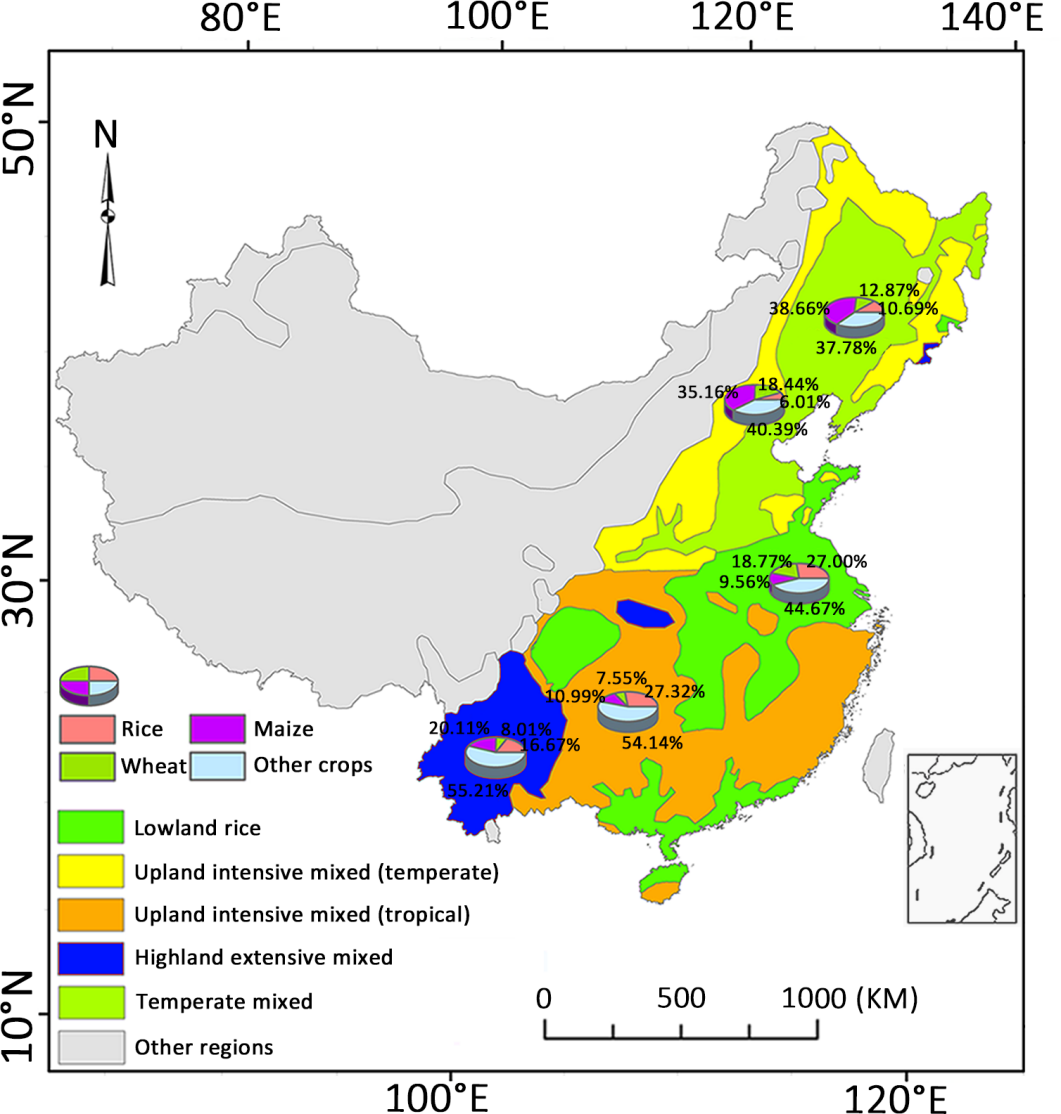
Rice, wheat, maize and other crop areas in five major cereal farming systems in eastern and southern China.

For this study, we identified the five most important farming systems in Eastern and Middle China for cereal (rice, wheat and maize) production (see Fig 1) based on the FAO/World Bank classification of 72 farming systems across six developing regions of the world [12]. These systems were the *lowland rice* system, *temperate mixed* system, *upland intensive system-temperate*, *upland intensive system-sub tropical*, and the *highland mixed* farming system. We split the FAO/World Bank *upland intensive* farming system into two sub-systems, the *subtropical-upland intensive* system and the *temperate-upland intensive* system, considering the wide range of latitudes and very different climates found within that system. About 93% of total national wheat, rice and maize production (% of national harvest area in Table 1) is produced within these farming systems (Fig 1). They are characterized by large human populations, high economic growth, and intensive-directed development strategies in agriculture and other industries [12].

**Table 1:** The distribution of types of yield trend across major cereals and farming systems in China, 1980–2010.

Farming system	% of national harvest area		Yield trend		
		CO	NI	SI	ST
		Rice			
Lowland rice system	59.7	0.2	4.7	22.3	32.5
Temperate mixed system	19.0	0.2	0.8	10.6	7.4
Upland intensive-temperate	2.2	0.0	0.1	1.2	0.9
Upland intensive-sub tropical	16.9	0.0	1.7	6.5	8.7
Highland mixed	1.6	0.0	0.0	1.2	0.4
System total (%)	99.6	0.4	7.4	41.8	50.0
System total (Harvest area, M ha)	29.3	0.1	2.2	12.3	14.7
		Wheat			
Lowland rice	51.6	0.3	7.7	37.3	6.3
Temperate mixed system	23.3	0.6	1.6	14.4	6.7
Upland intensive-temperate	9.1	0.4	2.4	4.9	1.4
Upland intensive-sub tropical	3.9	0.4	1.3	1.4	0.8
Highland mixed	1.1	0.1	0.3	0.2	0.5
System total (%)	89.1	1.8	13.3	58.2	15.8
System total (Harvest area, M ha)	21.2	0.4	3.2	13.8	3.8
		Maize			
Lowland rice	15.7	0.5	4.1	3.8	7.3
Temperate mixed system	62.9	0.7	13.7	7.2	41.3
Upland intensive-temperate	9.4	0.3	3.1	1.8	4.2
Upland intensive-sub tropical	4.8	0.0	0.5	3.4	0.9
Highland mixed	2.1	0.0	0.2	1.4	0.5
System total (%)	95.3	1.6	21.7	17.7	54.3
System total (Harvest area, M ha)	28.6	0.5	6.5	5.3	16.3

CO = yield collapsed, NI = yield not improved, SI = yield still increasing, ST = yield stagnated

The data used in this paper come from China's census bureau annual agricultural statistics for the period between 1980 and 2010. The data from 1980 to 2003 were collected from the *National Agricultural Statistics by County* and the data from 2004 to 2010 are from *the Provincial Statistical Yearbook* published each year. They track rice, wheat, and maize production and harvest area across the five cereal-dominated farming systems in China. The database comprised over 73000 census observations for thirty years in 2,463 counties. These data were converted into yield information at three variable spatial levels: the county, province/district/municipals and farming system. When a county pixel was segmented by more than one farming system, the crop harvest area of the county was broken into corresponding units weighted by spatial arable land allocation. The availability of census bureau data varied at the county level. Missing data values were common in some counties and years. For missing data in the body of the 30-year dataset, yearly values from preceding and succeeding years were averaged and used to interpolate the missing value. For those with missing data at the beginning or end of the time-period, an estimated trend from a contiguous county was used for the imputation. Data outliers with extremely high or low yield values (resulting from misreported data, gaps in survey statistics, weather fluctuations or pest infestation) were found in some counties and eliminated from the dataset (Data are supplied in the S1 Table to this paper).

### Yield trend analysis

**Fig 2:**
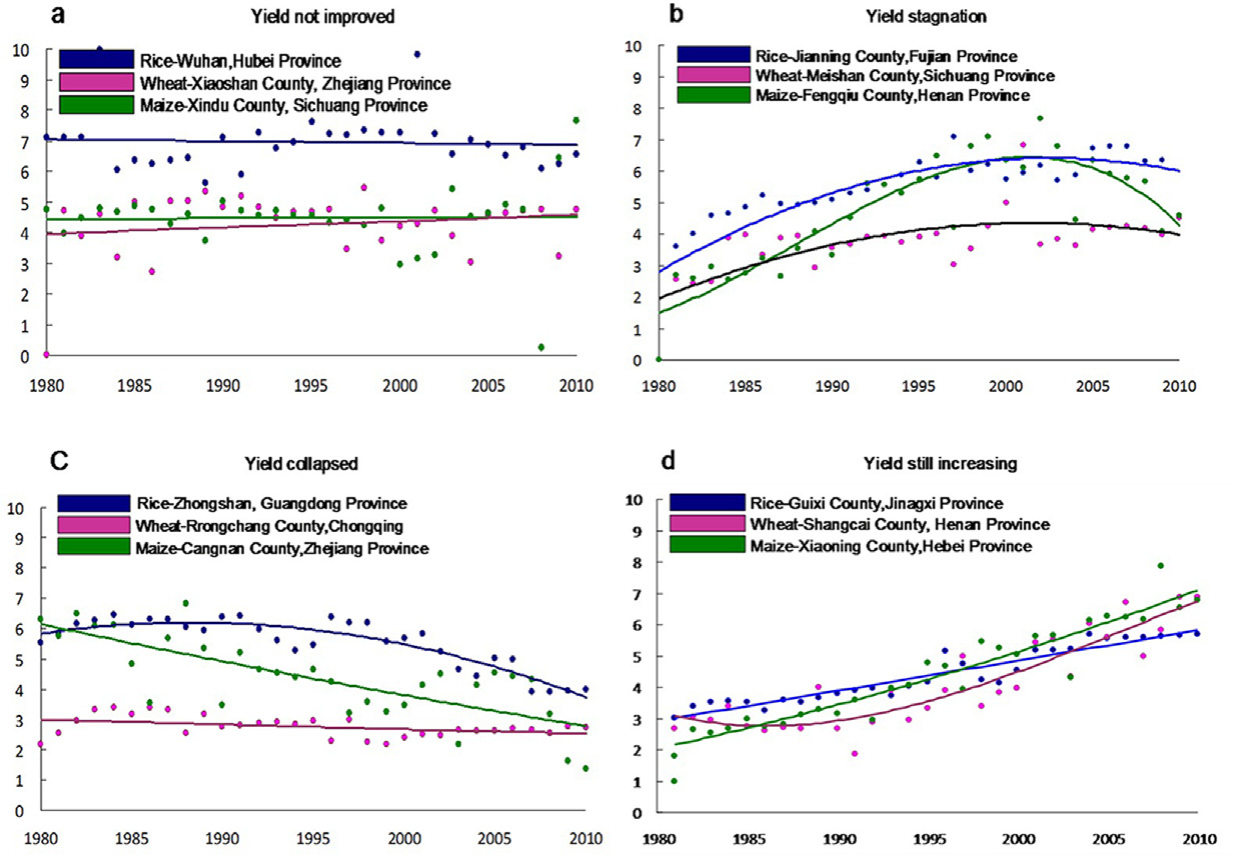
Examples of four types of cereal yield trends in China, 1980–2010. Y axis is grain yield in t/ha.

Yield trends were analyzed at each of the three levels of spatial unit (i.e. farming system, province and county) for each crop, through plotting observed yearly yield data between 1980 and 2010 and best-matching it with a regression model (either an intercept-only model, linear model or a quadratic model). Model parameters and curve characteristics guided the classification of yield trends into four categories that described the range of trends encountered (see examples in Fig 2). Similar types of yield trends were identified in a previous study by Ray et al. [13] and were recently used by Wei et al. [10]. The first type of yield trend was where yield had not improved (NI) during the 30-year period. This trend type was indicated by an intercept-only and linear model with positive slope and yield growth below 0.5 t ha^-1^ in the period (Fig 2A). Second, yield stagnation (ST), where yield improved earlier in the period but then yields stagnated or declined. In this case, the linear model showed a positive slope, the coefficients of the quadratic term in the quadratic model were negative, and the yield posted a maximum value which then held steady or declined (Fig 2B). Third, yield collapsed (CO), where yields have decreased since 1981, or initially increased and then collapsed to the level of the 1980s. This category exhibited a linear model with negative slope, the coefficients of the quadratic term in the quadratic model were negative, and the parabolic vertexes were before the middle of the period (Fig 2C). The fourth type, yield still increasing (SI), was characterized by increased yields during the period which continued to increase towards the end of the 30-year period. Positive slopes of the linear model with best fit R squares indicate the increase of yield. This type was identified by a quadratic model with negative coefficients and where the maximum values of yield (parabolic vertex) have not been reached to date, or positive coefficients with the parabolic vertexes near the ordinates (Fig 2D). Classification of these models is more complex with details included in the example Decision Tree as S1 Fig. Based on the rate of yield growth over the period, the crop areas within the SI category were further divided into three regions; those exhibiting “rapid growth” (the top 25% of yield growth rate in yield improving regions), “moderate growth” (the intermediate 50% of yield growth rate in yield improving regions), and “slow growth” (the bottom 25% of yield growth rate in yield improving regions).

The areas of yield trends for each specific crop in the farming system and province were summed from the county level data. Yield trends by county were imported into a geographical information system (GIS) dataset. These were matched to each grid (where each grid represents a county) in the GIS system and the areas of county-level yield trends were then aggregated into GIS units at the province and farming system level. Mapping allowed us to see where and how crop yields changed over time. To better understand the cereal food crop situation in major areas of China, a more detailed analysis of yield trends was carried out for each crop in their ten most important producing provinces. These results for the most important cereal producing areas helped us assess likely future cereal crop yield trends and their impacts on national food security.

### Climate-yield relation

As a changing climate may influence crop yield, we conducted multiple linear regression with rice and maize yields to test for linkages between climate change and crop yield trends. An approach based on first-difference time series for yield (ΔYield, yield on year to year change, the dependent variable) and growing season climate variables (ΔTmax, ΔTmin and ΔRf, representing the year-on-year change of maximum temperature, minimum temperature, and rainfall respectively, the independent variables), was used to estimate what effects a trend in the climate variables should have imposed on yield changes. This method has been often used in climate change and crop production studies [14–17]. Data on climate variables were collected from twenty-six China Meteorological Administration climate stations in the Northeast, twenty-seven in the *lowland rice system*, eight from North China, twenty from Huanghuaihai region, and thirty from South-west China. These data covered the thirty years from 1980 to 2010 (see S2 Table).

## Results

### Overall yield trends

**Fig 3:**
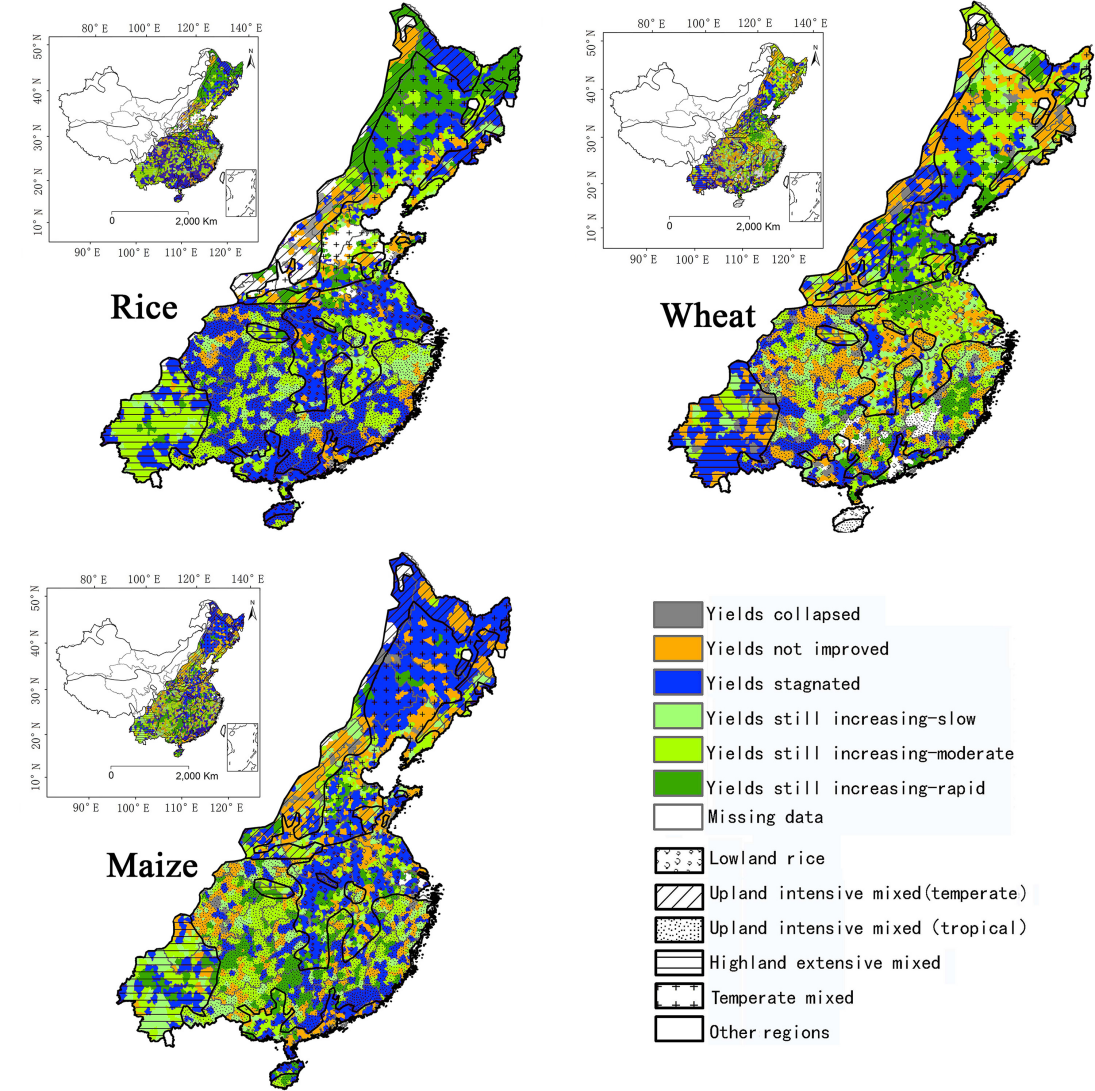
Maps of cereal crop yield trends (1980–2010) across major farming systems in China.

Our analysis shows that most of the cereal production area in the five farming systems (which together produced 93% of national cereal production) in China experienced significant yield growth between 1980 and 2010, with much of it in the early and mid-part of this time period. In recent years (the 2000s), yields on more than half of the areas in the five farming systems appear to have stagnated (around 14.7 million hectares for rice and 15.5 m ha for maize) (see Table 1 and Fig 3).

We found that rice yield continued to increase on 12.3 m ha, i.e. 41.8% of the harvest area. However, about 50% of the rice harvest area now shows yield stagnation (around 14.7 m ha) after a long period of increasing yield (Table 1). Yields collapsed or have not improved (with a slightly increase of 0.5 t ha^-1^) during the last decades on less than 2.3 m ha, or 7.8% of the total rice area.

Wheat yields have continued to increase on 13.8 m ha, i.e. 58.2% of the total harvest area (Table 1). Nearly 3.8 m ha (15.8% of the harvest area) exhibit yield stagnation. Furthermore, no improvement in wheat yield occurred over the thirty years on 13.3% of the harvest area, while yields collapsed on a small area (1.8%).

Since 2007, maize has become the most widely grown food crop in China followed by rice. However, in our analysis yields increased on only 17.7% of the maize harvest area (5.3 m ha), and have stagnated on a very large area (54.3%, 16.3 m ha) after some improvement early in the time period (Table 1). Additionally, yields have not improved at all on 6.5 m ha (21.7% of the maize area) and have collapsed on 0.5 m ha (1.6%).

### Yield trends across farming systems and provinces

Within the five farming systems, only 7.8% of rice growing areas saw no yield increase over the 30 years. These areas were found mostly in the *lowland rice* system, marginal areas in Sichuan province, and parts of the *upland intensive* system in Shanxi, Shaanxi and Inner Mongolia (Fig 3). The *lowland rice* system is extremely important for rice production with 59.7% of the national rice harvest area. In this system, 22.3% of the national rice area is still experiencing an increase in yield, but more (32.5% of the national rice area) has stagnated. The *temperate mixed* system has 19% of the national rice harvest area, with half of it experiencing yield growth (mostly of the ‘rapid-increasing’ type), and 7.4% of total stagnated rice area is found here. The *sub-tropical upland* system has 16.9% of national rice harvest area and half (8.7%) has stagnated. In Fig 3, large blue pixels show the large areas where rice yields have stagnated in southern farming systems of China, including the *lowland rice*, *upland intensive-sub tropical*, and *highland mixed* systems. The large areas with moderate yield increases are shown in bright green. For the northern *temperate* and *upland intensive-temperate* systems, located mostly on the Northeast Plain of China, the areas where rice yields are rapidly increasing (marked in dark green) covers over half of the area (Fig 3). Rice yields have collapsed on less than 1% of the rice harvest area, mostly located in the remote mountains of Shaanxi and Shanxi.

Wheat yields did not improve between 1980 and 2010 on 13.3% of harvest areas in the farming systems. Of this, 7.7% of the area was found in the *lowland rice* system, which is the most important wheat growing system with 51.6% of China's wheat harvest area. In the *lowland rice* system, 37.3% of the national wheat area has experienced increased yield, with another 6.3% stagnated. *The temperate mixed* system has the second largest wheat harvest area (23.3% of national total). Here more than half (14.4% of the national total) had increasing yields, with 6.7% stagnated. Yields increased on 72.3% of the wheat area in the *lowland rice* system and 61.7% of the wheat in the *temperate mixed* system. In the highly productive wheat growing areas, wheat yields continue to increase rapidly on the Huabei plain, in Hebei, Henan, Anhui and Shandong provinces (Fig 3) of the *temperate mixed* system. These are major parts of the most productive wheat zones.

Maize yields did not improve between 1980 and 2010 in important remote upland landscapes, including mountain areas in Shanxi, Shaanxi, and Sichuan provinces, which comprise 21.7% of the maize harvest area in China. In the *temperate mixed* system, which is China’s major maize growing area (comprising 62.9% of the national maize harvest area), yields have stagnated in over 65% of that system. These comprised 41.3% of the national maize area with stagnated yields. Only 11.5% of the maize area (7.2% of national maize) had yields that have continue to rise. Yields have also stagnated in the *lowland rice* and *upland intensive-temperate* systems, where 46.4% (7.3% out of 15.7%) and 44.7% (4.2% out of 9.4%) of maize harvest areas have stagnated yields. In contrast, in the southern production systems, maize yields have continued to trend upwards, even in those systems with a small share of maize production. In the *upland intensive-sub tropical* and *highland mixed* systems, 70.8% (3.4% out of 4.8%) and 64.9% (1.4% out of 2.1%) of the maize growing areas experienced yield increases. Rapid and moderate rates of increase in maize yields were found in Hunan, Guangxi, Sichuan, and Yunnan provinces (Fig 3).

### Yield trends in Top 10 provinces

**Fig 4:**
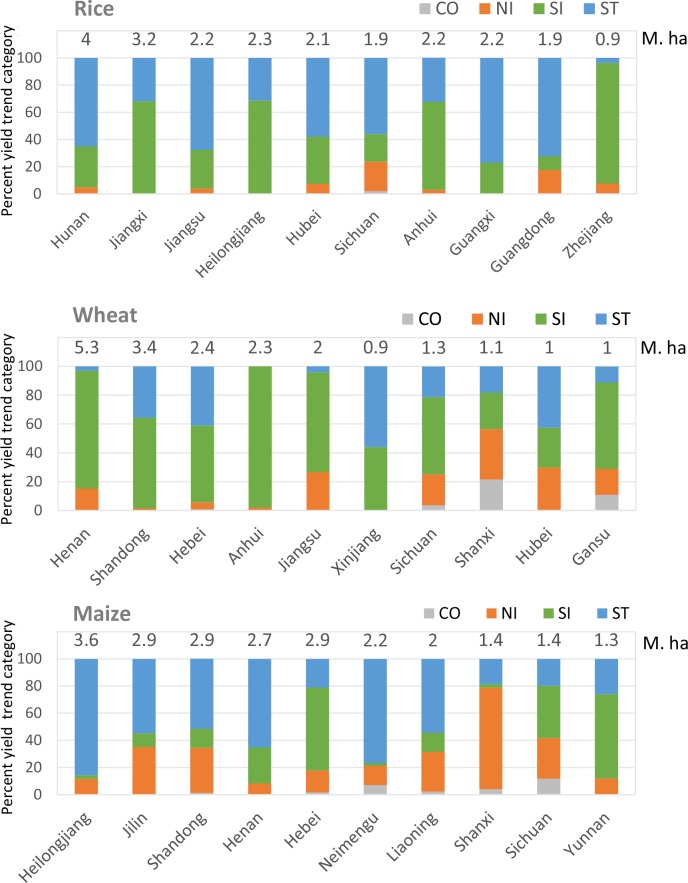
Crop yield trend status for top 10 producers in China. The top 10 producers were derived from average crop production of 2006–2010 report by NSB, arranged in the horizontal coordinate by order of production size. Overall area was based on the harvest area from the five focus farming systems. Harvest area based on average 2006–2010.

The top ten rice producing provinces are located in central and southern China, with the addition of Heilongjiang province in the northeast. Yields have stagnated in some of these ten provinces, but have risen in others. Six provinces are experiencing yield stagnation on more than half of their rice growing area. The most widespread stagnation has happened in Guangxi province where 77% of the harvest area has stagnated and in Guangdong with 72.3%. Hunan and Jiangsu, the first and third largest producers, have seen stagnation on 67.6% and 65.0% of their rice areas, along with 58% in Hubei and 56% in Sichuan (Fig 4). However Zhejiang, Jiangxi and Anhui provinces (which are located in central and eastern China), have seen yields increase on 88.7%, 68.1% and 64.5% of their harvest areas. Heilongjiang province (in China's far northeast) recorded yield increases on 68.7% of its area.

The most advantaged wheat producing provinces are located north of the Yangtze River in North China. The top ten producers showed relatively consistent trends to increased yield, with seven provinces experiencing a yield increase on more than half their harvest area. Yields have risen on up to 98% of the wheat harvest area in Anhui province, 81.8% in Henan, 69.3% in Jiangsu, 63.0% in Shandong, 59.9% in Gansu, 53.6% in Sichuan and 53.2% in Hebei. The remaining provinces among the ten wheat producers are Xinjiang and Shanxi, and Hubei province, where less than half of the harvest area showed increased yields.

Maize is grown predominantly in Northeast China, especially in Heilongjiang, Jilin and Liaoning provinces; and in North China, principally in Shandong, Henan and Hebei provinces. Our analysis suggests that all the above producer provinces have experienced a stagnation of maize yield on over 50% of their harvest area, except for Hebei. Among them, the largest producer, Heilongjiang, had 85.5% of its maize area with stagnated yields. The second and third largest producers, Jilin and Shandong, witnessed stagnation on 54.8% and 51.6% of their maize areas. The fourth producer (Henan), had 65.3% stagnated, along with 54.3% for Liaoning. Thus our assessment indicates a widespread stagnation of yields in China’s traditional maize regions. In contrast, other producer provinces located in the south and west of China have shown continued growth in maize yield. Sichuan and Yunnan are dominated by upland landscapes, where rice, wheat and maize all grow well. Maize is becoming more important in those two provinces, with a 38.5% increase in maize yield on a 62.1% larger harvest area over the period 1980 to 2010.

### Climate effect

Climate change results are in Tables 2 and 3. ΔTmin was positively related (*p* < 0.05) to rice yield change, and ΔRf was negatively related (*p* < 0.05) in Northeast China, the temperate rice production region, where effective accumulated temperature is a key factor for rice growth. Temperature and rainfall were found to be unrelated to change in rice yield in the *lowland rice* system. For maize, ΔTmax was significantly related (*p* < 0.05) to maize yield changes in all three temperate maize growing regions, where an increase in Tmax reduced yield. With ΔTmin there was a significant positive relationship between Tmin and maize yield change, with higher Tmin giving higher maize yield in Northeast China. ΔRf was negatively related to maize yield change in the Northeast and in Huanghuaihai region. In the tropical upland area in south west China, changes in maize yield were not significantly related to temperature and rainfall variables.

**Table 2:** Summary results of multiple linear regressions based on rice first difference time series data (1980–2010) for two dominant rice production areas in China.

Region	Coefficients	S.E	t Stat	P-value	Lower 95%	Upper 95%
**Northeast China**						
Intercept	0.1433	0.0636	2.2537	0.0245	0.0185	0.2682
ΔTmax	-0.0077	0.0106	-0.7284	0.4666	-0.0285	0.0131
ΔTmin	0.0247	0.0118	2.0891	0.0371**	0.0015	0.0479
ΔRf	-0.0009	0.0003	-3.1064	0.0020***	-0.0014	-0.0003
R Square	0.0176	Sign F	0.0064			
**Lowland Rice**						
Intercept	0.1062	0.0588	1.8048	0.0717	-0.0094	0.2217
ΔTmax	0.0017	0.0090	0.1877	0.8512	-0.0159	0.0193
ΔTmin	-0.0141	0.0119	-1.1859	0.2362	-0.0375	0.0093
ΔRf	0.0000	0.0001	-0.0380	0.9697	-0.0002	0.0002
R Square	0.0037	Sign F	0.5871			

** significant at 0.05 level

*** significant at 0.01 level

**Table 3:** Summary results of multiple linear regressions based on maize first difference time series data (1980–2010) for three dominant maize production areas in China.

Region	Coefficients	S E	t Stat	P-value	Lower 95%	Upper 95%
**Northeast China**						
Intercept	0.0732	0.3632	0.2017	0.8402	-0.6398	0.7863
*ΔTmax*	-0.2406	0.0613	-3.9276	0.0001***	-0.3609	-0.1203
*ΔTmin*	0.2162	0.0712	3.0376	0.0025***	0.0765	0.3560
*ΔRf*	-0.0039	0.0018	-2.2318	0.0259**	-0.0074	-0.0005
R Square	0.0207	Sign F	0.0015			
**North China**						
Intercept	0.0576	0.0727	0.7926	0.4288	-0.0856	0.2008
ΔTmax	-0.0251	0.0114	-2.1993	0.0288**	-0.0475	-0.0026
ΔT*min*	0.0226	0.0151	1.5003	0.1349	-0.0071	0.0523
ΔRf	-0.0005	0.0004	-1.4408	0.1510	-0.0012	0.0002
R Square	0.0209	Sign F	0.1716			
**Huanhuaihai**						
Intercept	0.1004	0.1135	0.8838	0.3772	-0.1228	0.3235
ΔTmax	-0.0490	0.0155	-3.1650	0.0017***	-0.0795	-0.0186
ΔT*min*	0.0364	0.0205	1.7733	0.0768	-0.0039	0.0768
ΔRf	-0.0003	0.0002	-1.9982	0.0463**	-0.0006	0.0000
R Square	0.0223	Sign F	0.0139			
**Southwest China**						
Intercept	0.0894	0.0532	1.6790	0.0939	-0.0153	0.1940
ΔTmax	0.0089	0.0112	0.7935	0.4279	-0.0132	0.0310
ΔT*min*	0.0035	0.0124	0.2814	0.7786	-0.0209	0.0278
ΔRf	-0.0001	0.0002	-0.3342	0.7384	-0.0004	0.0003
R Square	0.0089	Sign F	0.2847			

** significant at 0.05 level

*** significant at 0.01 level

## Discussion on the Implications of Yield Trends for China’s Food Security

### Yield trends and areas

According to the overall yield trends in our study, all three cereal crops have continued to show yield increases on large parts of their areas in China over the period 1980–2010. Yield declines are uncommon for all three crops but yield stagnation and no improvement in yield are now widespread. In general, our findings on yield trends and areas are consistent with a previous study by Wei et al. (2015) [10]. Our results were especially similar to those of Wei et al. for rice. For wheat, our results showed a higher proportion of the harvest area with increasing yields (58.2%) than Wei (49.4% of counties), and far less area stagnated (15.8% harvest area) than the 42% of counties with stagnation reported by Wei et al. For maize we calculated that a very large 54.3% of the area is suffering from yield stagnation, compared with 42.3% of counties in Wei et al. Differences between the two studies are likely to be due to the different statistical unit employed (our work aggregated counties with the same yield trend by crop area while Wei et al summed the number of counties with each yield trend). Additionally Wei incorporated counties from all of China, including vast areas of sparsely-cropped western China, while we concentrated on the major cereal areas of northern, eastern, central and southern China. This may explain why results were particularly different for wheat, where a significant portion of stagnated counties in western for wheat are stepped in to Wei’s statistic.

Considering the spatial distribution of types of yield trend, our findings complement Wei et al. For rice, large areas in southern farming systems show yield stagnation (Fig 3) as seen for southern counties in Wei’s map. For wheat, areas with stagnated yields are widely distributed and areas with increasing yields are concentrated in central and northeastern parts of China in both studies. With maize, we found yield stagnation to be more broadly distributed in our map than Wei’s, especially in northeast areas such as Heilongjiang, Jilin and Liaoning, which are mostly in the temperate farming system. Increasing maize yields are centralized in southern farming systems, i.e. the *upland intensive-sub tropical* and *highland mixed* systems, while the yield increasing counties in Wei et al. were found more widely in the traditional northern maize producing region. In the Northeast of China, maize yields have stagnated at relatively high yield levels, at round 6 t/ha, after a long period of intensifying cultivation. To sustain the environment and natural resources, the Government of China has recently proposed policies to reduce the maize planted area and promote land fallowing and crop rotation in those regions [18].

### Cereal demand

China shows many of the characteristics of the emerging global food crisis. Continued expansion of demand for food (expanding human population and diet changes towards more meat and dairy products) combine with tighter supply as increases in energy needs across the country, the loss of agricultural land and environmental degradation and climatic changes all reduce the potential for food production [19–22]. China can help to reduce these pressures and contribute to global food security if she can meet her food crop self-supply target of 95%. However there has been a recent trend to increase the net import of staple food crops including maize, wheat and soybeans in China [23,24]. Due to adverse weather and competitive international prices in 2012, China imported 5.2 m t of maize and 3.7 m t of wheat, which were the highest recorded imports in Chinese history. In 2014, China imported 2.6 m t of maize and 2.97 m t of wheat, and imports of these crops are projected to each reach 4 m t in 2024 [25]. Such large imports might undermine and distort global food availability and prices in international markets contributing to food insecurity in vulnerable parts of the world. The question is how China can meet more of the increasing demand through local production when recent crop yield trends are insufficient and large crop areas in the most important farming systems show stagnated or declining yield.

### Production constraints

Given that around half of China's grain croplands now show stagnated yields, it is important to identify and address the production constraints that have resulted in the yield stagnation. Systematic studies show that mixtures of numerous biotic, abiotic, socio-economic and crop management factors constrain yields [26–28]. For wheat, an average grain yield on farm was estimated to be above 5.8 t/ha in the *temperate mixed* system, with a relatively large farm grain yield gap of 3.1 t/ha due to many constraints including lodging, heat during grain fill, mid-season' drought (crop water deficit), terminal (grain filling) drought, irrigation problems and poor management of N fertilizer [27,29]. The *temperate mixed* system comprises some extremely productive wheat farming environments in which farmers use large amounts of inputs and management to obtain high yields. We found little yield stagnation in this system and wheat yields have continued to increase on nearly 62% of the area (Table 1). Wheat is also widely planted in the *lowland rice* system where the current yield is around 5.1 t/ha, and yields continue to increase on 72.3% of the wheat area (Table 1). Here poor seedbed preparation, poor management of N fertilizer, N fertilizer expensive and short supply, and N deficiency were considered severe constraints.

We found that stagnation of rice yields is now common in the main Chinese rice growing regions of the *lowland rice* and *sub-tropical upland* systems. In these areas, rice fields have been intensively cropped for very long periods of time. It was reported that the most severe constraints in these areas included deficiencies of potassium, phosphorus and nitrogen, cold waterlogged soils, cold at anthesis, low soil organic matter and drought. Inappropriate nutrient/fertilizer use and management, soil fertility depletion, the low price of output/products, and leaf and stem pests’ were responsible for reduced yields of rice in the *lowland rice* system. In the *upland intensive-subtropical* system, leaf and stem pests was considered the most important constraint, with drought or intermittent water stress, inadequate water management and inadequate farmer knowledge/training among other major constraints [27,28].

In the most important maize system, the *temperate mixed*, we found widespread yield stagnation (Table 1). Drought was assessed to be the top constraint for maize in both the *temperate mixed* and *lowland rice* systems [26]. Apart from drought, pests (borers, moths and bollworm) and a wide range of blights, smut, and rots affect maize production in the *temperate mixed system*. Low soil fertility was cited as a major constraint for maize in the *lowland rice* system, along with diseases and flooding.

### Changing climate

Climate change and its impact on food security is a key focus and challenge for China this century [30]. Recent research has assessed climate change and its likely impact on crop yield changes in China [17,31]. Rising temperatures, altered rainfall patterns, and more frequent extreme weather events will affect crop production in complex ways, often in those places that are already most vulnerable [32]. The average temperature has increased by 1.2°C since 1961 in China [33], precipitation has declined in the already dry North and more and variable rainfall is expected in the wet South [34].

In our climatic assessment we selected rice and maize for testing since yield trends with these crops exhibit large differences within areas and farming systems while, in contrast, wheat yields are increasing among most wheat producers. From our study it seems the overall impact of climate change on these crops in China is uncertain, with some benefits and some damage expected. The increasing ΔTmin in the Northeast should contribute to higher rice and maize yield, while increasing ΔTmax may negatively affect maize yield in temperate systems. These findings are supported by previous studies. Rice yields in the northeast appear to have increased by 4.5–14.6% per 1°C in response to night-time warming between 1951 and 2002 [17]. A 4.5% reduction in average wheat yields can be attributed to rising temperatures over the period 1979–2000 [31]. However, other research has shown conflicting results. Peng et al. (2004) calculated that a 25% reduction of the global rice yield was due to warming temperatures [35]. The average impacts of higher temperatures and more rainfall are positive in China [36]. Current researches based on panel data at province scale indicate that marginal increases in temperature and rainfall have very different effects on grain production in different regions with seasonal effects.

### Limited arable land and property rights

Since the 1950s, China’s population has doubled to 1.3 billion while the total arable land has increased by less than 30% to about 121.7 m ha [37]. Further increases in the cropping area would offset part of the impact of yield stagnation, but there is little scope for that in China. China is using almost all the available arable land [33], and agricultural land continues to be lost to accelerating urbanization [38]. Arable land *per capita* is currently just 0.1 ha and this will fall. Cultivation based on the *Household Contract Responsibility System* brought huge improvements in productive potential, promoted food production and contributed to the reduction of rural poverty during the early reform period in the 1980s and 1990s, but resulted in a small farm size, estimated to now average 0.53 ha per farm household in China. Many aspects of modern farming technology and cultivation techniques are difficult to employ on small farms, which constrains crop yields and limits the improvement of income for most farm households [39]. Reforms to the market for farmland and land property rights are ongoing, which aim to increase China's farm size and then promote agricultural productivity [40].

The non-agricultural sectors in China are developing quickly and have enrolled many young workers from rural areas. There has been a large shift of labor out of agriculture, and this trend continues. The size of the transfer of rural labor to urban areas increased to more than 250 million people in the year 2011 [41], which is around 38% of the total rural active labor force. Thus it is essential to coordinate rural labor migration and food security.

Farmers that remain in agriculture are mostly under-educated and older generation and this is a further challenge to develop farming and farm productivity. Previous experience from developed countries showed that an emphasis on the education of remaining farmers and agricultural workers was important in promoting agricultural productivity and in adopting new tools, varieties, methods and technology in agriculture [42]. As an example, in the USA, the proportion of all labor in the agricultural sector fell to 10.8% by 1970, and reduced further to less than 3% in the 1990s. During this time the agricultural output value of the USA continued to rise. For China, new energetic and well-prepared business-smart farmers and modern agricultural enterprises are needed for the more information- and market-driven environment.

### Water shortage

Reduced availability and uneven distribution of water are extremely serious in China, and these have been considered the biggest threat for livelihoods and food security [43]. In the northern *temperate mixed* system, where wheat and maize are the dominant crops, and where maize now exhibits significant yield stagnation, water shortage and drought are the major concerns in crop production. The north of the country, holding nearly half the total population of China and 65% of the arable land, has only 18% of the total water resource. In many areas, agricultural production is now highly dependent on groundwater, which together with more intensive use of water for industry and daily life has resulted in an unsustainable decline in the water table of around 40 cm each year [44, 45].

Comparatively, in the south where rice is the key crop, rainfall (especially summer rainfall) is often more than sufficient and flooding of crops is common. In central areas such as the middle and lower areas of the Yangtze River Valley (where water resources are considered abundant), seasonal and regional drought and water shortages still occur. Water security is the foundation of food security. To realize the crop yield potential, there is a need to strengthen water conservancy facilities and improve water use efficiency combined with South-to-North water diversion and grain transportation.

### Fertilizers and pollution

In recent years, as arable land has reduced and the shortage of water become more serious, environmental degradation has received more attention in China. Intensive cultivation using more inputs has resulted in low nutrient use efficiencies and environmental pollution [46]. China is currently the largest consumer of chemical fertilizer and pesticide in the world [47]. Recently, the average amount of N fertilizer applied on food crops in China was estimated at 190 kg ha^−1^ [48]. With the increased and irrational use of chemical fertilizer, fertilizer use efficiencies have decreased rapidly [49,50]. Most Chinese farmers fertilize based on habit rather than on-site soil nutrition status and crop nutrient requirements, and much of the excess fertilizer is lost to the environment, leading to degradation of soil and water quality [51,52]. It is estimated that sixty percent of Chinese lakes are facing eutrophication, often the result of fertilizer leaching and runoff from agricultural activities adversely affecting the irrigation resource and drinking water [33].

## Ways Forward

It is likely that the widespread use of appropriate technology innovations will continue to alleviate cereal production constraints, raise yields and help China to increase agricultural productivity, especially if water and land property rights can be addressed, and environmental degradation contained. Since there is little scope to increase the cropping area, China will need to ensure continued cereal yield increase in regions with recent growth in yield, without further damaging the environment, by employing sustainable intensification approaches to crop management [40]. Optimization of land use in different parts of the country by encouraging farmers to plant the best type of cereal crop and varieties for their area (based on resource availability, yield performance and benefit-cost situation) should help in the short-term. This has happened in the past where the focus for national rice production shifted around 340 kilometers from the South to the North since the 1960s so that the Northeast of China is now a significant new rice producer [8]. From the 1990s, the overall rice area planted in China was slowly reducing, while the expansion of new rice areas with increasing yield contributed greatly to maintain rice production in the Northeast (Fig 3, green area in Northeast) where the original dominant crops included maize and soybean.

For the future, prospects appear brightest for maize. Maize has become the biggest crop in China by planted area since 2007 and by production since 2012. There is great potential to raise maize production by encouraging more planting in central and southern China. Demand for maize is expected to continue to rise associated with increasing population and more need for animal feeds to produce high protein human foods. Most of the widespread stagnation of maize yield shown in this study has occurred in the northern *temperate mixed* system where yields are already high. However in the Southwest production region, including the *highland mixed* system, yields continue to rise on most of the maize area, although the maize harvest area is smaller and yields lower. Maize evolved in sub-tropical regions and spread to temperate areas, so it has wide adaptability and can be planted across low and high latitudes. If maize area and production can be expanded in the large parts of the south that have increasing yield trends this would substantially raise maize output. These areas have favorable rainfall for mixed rainfed and irrigated cropping that can respond well to other inputs, especially fertilizers.

In conclusion, crop yield improvement is necessary to feed the expected 1.6 billion Chinese in the near future. However land, water, and environment limitations continue to undermine agricultural productivity and threaten future food security. Improved crop management, and policy innovations must promote crop yields and the sustainable use of agricultural resources to achieve continued food security. There are good prospects that a better matching of the type of cereal crop and varieties to soil and climate in China will expand the areas with potential for further improvement of yield to better meet the demand for food in coming years.

## Supporting Information

S1 Fig Decision tree and example. (PDF)

S1 File Permission for the copyright of Figs 1 and 3. (PDF)

S1 Table Raw yield data 1980–2010. (XLS)

S2 Table Climate data 1980–2010. (XLSX)
