# Supplementary material for: Patterns of Cereal Yield Growth across China from 1980 to 2010 and Their Implications for Food Production and Food Security
Source: PLoS One. 2016 Jul 12;11(7):e0159061. doi: 10.1371/journal.pone.0159061 (PMC4942084; doi:10.1371/journal.pone.0159061)

S1 Fig. Decision Tree and Example (We take Fig 2b Maize-Fengqiu County, Henan Province as an example for addressing the yield trend category classification. Firstly we draw scatter plotting of 30 years' yield from this county in EXCEL, then use intercept-only model, linear model or a quadratic model to match the trend, as attached. According to the decision criteria stated in above tree (the linear model showed a positive slope, K>0, the coefficients of the quadratic term in the quadratic model were negative, a<0, and the yield posted a maximum value which then held steady or declined, $10 < \frac{b}{2a} < 30$), we justify this trend as Stagnation.)

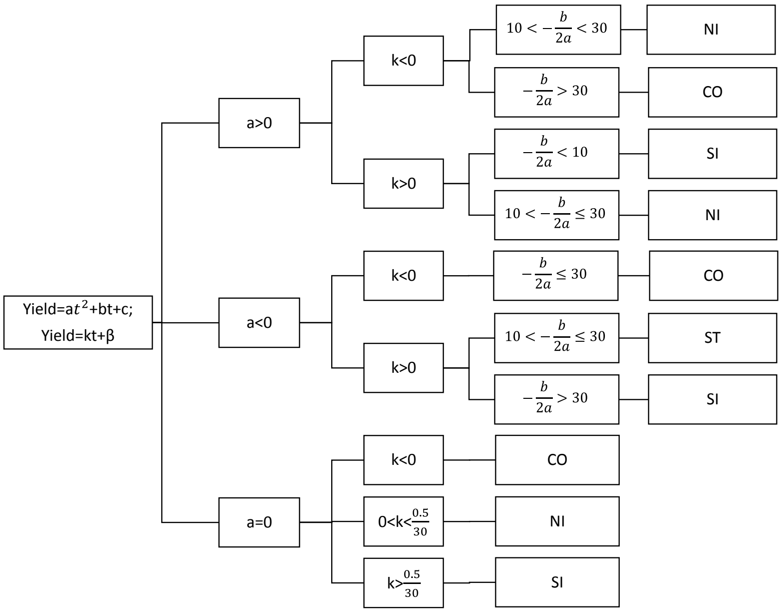

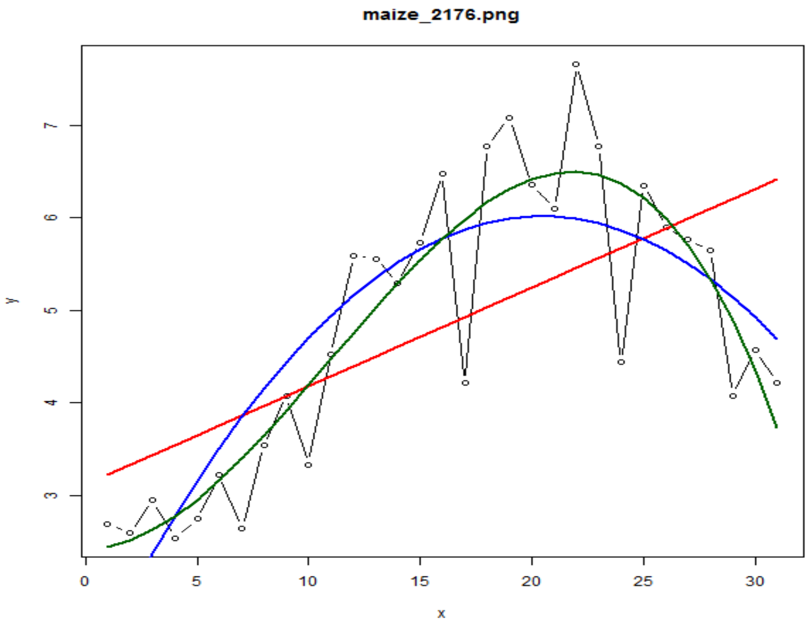

Supplement: S1 Fig — (PDF) [file pone.0159061.s001.pdf]
