# Supplementary material for: Patterns of Cereal Yield Growth across China from 1980 to 2010 and Their Implications for Food Production and Food Security
Source: PLoS One. 2016 Jul 12;11(7):e0159061. doi: 10.1371/journal.pone.0159061 (PMC4942084; doi:10.1371/journal.pone.0159061)
Supplement: S1 File — (PDF) [file pone.0159061.s002.pdf]

Permission for the copyright of Figures 1 and 3.

Refs: PONE-S-16-09081
Patterns of cereal yield growth across China from 1980 to 2010 and their implications for food production and food security

We are writing to permit the open-access journal PLOS ONE publish the figures 1 and 3 in referred paper under CCAL. Those two figures were originally made by the authors in 2016.

Sincerely,

Li Xiaoyun

Xiaoyun Li et al

PS: The authors are Li, Xiaoyun; Liu, Nianjie; You, Liangzhi; Ke, Xinli; Liu, Haijun; Huang, Malan & Waddington, Stephen R. All the authors acknowledge and agree to this permission.
